# The Roles of FGF21 and ALCAT1 in Aerobic Exercise-Induced Cardioprotection of Postmyocardial Infarction Mice

**DOI:** 10.1155/2021/8996482

**Published:** 2021-11-05

**Authors:** Wenyan Bo, Yixuan Ma, Yue Xi, Qiaoqin Liang, Mengxin Cai, Zhenjun Tian

**Affiliations:** Institute of Sports and Exercise Biology, Shaanxi Normal University, Xi'an 710119, China

## Abstract

Aerobic exercise mitigates oxidative stress and apoptosis caused by myocardial infarction (MI) even though the precise mechanisms remain completely elusive. In this study, we investigated the potential mechanisms of aerobic exercise in ameliorating the cardiac function of mice with MI. In vivo, MI was induced by left anterior descending (LAD) coronary artery ligation in wild-type mice, *alcat1* knockout, and *fgf21* knockout mice. The mice were exercised under a moderate-intensity protocol for 6 weeks at one week later post-MI. In vitro, H9C2 cells were treated with lentiviral vector carrying *alcat1* gene, recombinant human FGF21 (rhFGF21), PI3K inhibitor, and H_2_O_2_ to explore the potential mechanisms. Our results showed that aerobic exercise significantly increased the FGF21 expression and decreased the ALCAT1 expression in the hearts of mice with MI. *fgf21* knockout weakened the inhibitory effects of aerobic exercise on oxidative stress, endoplasmic reticulum (ER) stress, and apoptosis in mice with MI. Both/either *alcat1* knockout and/or aerobic exercise improved cardiac function by inhibiting oxidative stress and apoptosis in the MI heart. rhFGF21 inhibited both H_2_O_2_ and overexpression of ALCAT1-induced oxidative stress and apoptosis by activating the PI3K/AKT pathway in H9C2 cells. In conclusion, our results showed that aerobic exercise alleviated oxidative stress and apoptosis by activating the FGF21/FGFR1/PI3K/AKT pathway or inhibiting the hyperexpression of ALCAT1, which ultimately improved the cardiac function in MI mice.

## 1. Introduction

Heart failure (HF) is a major public health problem threatening the health and safety of over 23 million people worldwide. Myocardial infarction (MI) is one of the important inducements of HF with a mortality rate close to 5% a year and the incidence rate and mortality rate younger and rising [1]. There is a growing clinical consensus that exercise training after MI can increase the quality of life and prevent future complications and longevity in infarcted patients [2]. Therefore, it is very important to find the effective-targeting molecules of exercise rehabilitation after MI.

Fibroblast growth factor 21 (FGF21), as an endocrine factor, is a unique member of the FGF superfamily and belongs to the subgroup of fibroblast growth factor 19. It is involved in the regulation of metabolic process and has the effects of antioxidative stress and apoptosis [3–6], anti-inflammatory, and promotion of angiogenesis [6, 7]. Oxidative stress and endoplasmic reticulum (ER) stress induce apoptosis as a critical pathophysiological mechanism for cardiomyocyte injury. Many studies have reported that FGF21 significantly inhibited oxidative stress and ER stress-induced apoptosis in damaged myocardium [6, 8, 9]. FGF21 inhibits ER stress-induced cardiomyocyte apoptosis by activating the FGFR1/PI3K/AKT pathway [4]. Additionally, clinical experiments have shown that the content of FGF21 in the blood of healthy subjects increased significantly after exercise [10, 11]. Animal experiments have found that the level of serum FGF21 increased significantly after a single acute exercise in mice [12]. These indicate that FGF21 might play an organ protective role as “exerkines.” However, little is known about whether FGF21 mediates the cardioprotective effects of aerobic exercise on MI and whether the mechanisms are involved in the oxidative stress and ER stress-induced apoptosis.

Cardiolipin (CL) is highly sensitive to oxidative stress injury and oxidized in the early stage of apoptosis [13–15]. Lysocardiolipinacyltransferase-1 (ALCAT1), located in ER, is the key enzyme of CL remodeling. Under pathological conditions, the abnormal increase of ALCAT1 leads to the massive production of ROS, mitochondrial dysfunction, and insulin resistance [16–18], which is the core link of obesity [19], Parkinson's disease [20], diabetes [21], and other chronic diseases and aging process [22]. Furthermore, ALCAT1-targeted inactivation prevents diet-induced obesity and nonalcoholic fatty liver disease (NAFLD) and neurotoxin1-methyl-4-phenyl-1,2,3,6-tetrahydropyridine- (MPTP-) induced neurotoxicity, reduces oxidative stress injury, improves mitochondrial function, and mitigates apoptosis [19, 20, 23]. Exercise reduces the expression of ALCAT1 in the skeletal muscle of mice [24]. Aerobic exercise alleviates oxidative stress-induced apoptosis in the kidneys by inhibiting ALCAT1 in MI mice [25]. However, whether ALCAT1 is involved in the effect of cardioprotection of exercise in the post-MI heart has not been elucidated.

In conclusion, we used wild-type (WT) mice, *fgf21* knockout mice, and *alcat1* knockout mice, as well as H_2_O_2_, lentiviral vector carrying *alcat1* gene, and recombinant human FGF21 (rhFGF21) in H9C2 cells, to explore these problems. This study will provide a theoretical basis and experimental support for the prevention and rehabilitation of ischemic heart disease by aerobic exercise and further elucidate the potential protective mechanism of aerobic exercise on ischemic heart.

## 2. Materials and Methods

### 2.1. Animals and Exercise Protocols

30 wild-type (WT) male C57/B6J mice (20 ± 5 g, 8-weeks old) were purchased from the Laboratory Animal Centre of Xi'an Jiao Tong University (Xi'an, China; animal breeding license number: SCXK (Shaan) 2012-003). *alcat1* heterozygous mice knocked by CRISPR/Cas9 were obtained from Cyagen Biological Co. Ltd (China), propagated to the fourth generation, and homozygous selected for the experiment. Male *alcat1^−/−^* mice were screened by the mice tail identification method (Fig. S1). The Cre/loxP recombination system was used to conditionally knockout the loxP^+^/^+^ mice of *fgf21*, (Jackson Laboratory, USA). The genotype of F2 generation after propagation and expansion was identified; then, the tail vein was injected with the adeno-associated CRE virus (rAAV-CMV-EGFP-P2A-CRE-WPRE-bGHpA, AAV2/9, titer: 5.73E+12vg/ml) to the loxP^+^/^+^ mice of *fgf21* (Fig. S2). All experiment designs and surgical operations were performed in compliance with the guidelines for the use of experimental animals and have been approved by the Ethical Committee of Shaanxi Normal University.

The MI model was established by permanent ligation of the left anterior descending coronary artery (LAD), the ligation sites were located 2 mm below the junction of the left atrial appendage and pulmonary conus. Echocardiography was performed on all mice undergoing MI surgery on the third postoperative day to determine modeling success, and mice with EF values of 40-55% were selected for follow-up experiments. WT mice were randomly divided into three groups (*n* = 10): sham surgery group (S), MI myocardial infarction group (MI), and six-week exercise training with MI group (ME). The grouping of *alcat1^−/−^* mice was the same as that of WT mice (*n* = 10). *fgf21^−/−^* mice were divided into the post-MI six-week exercise training group (*fgf21^−/−^* ME).

Exercise-trained animals were subjected to six weeks of exercise on a motorized rodent treadmill (Model ZH-PT, Anhui Zhenghua Technology Co., China) from the second week after induction of MI. The first week was adaptive training, the mice ran 60 min daily from a speed of 5 m/min to 10 m/min. Formal training was performed later, 10 min with 8 m/min in speed and 55 min with 10 m/min in speed each day, 5 d/week for six-week. The death of two mice was caused by this exercise protocol during the entire study. After the training, the cardiac function of mice was detected by echocardiography and then decapitated. The blood and heart were collected quickly and put into formaldehyde or liquid nitrogen for subsequent experiments.

### 2.2. Measurement of Echocardiographic and Histological Staining

The cardiac physiological functions were evaluated by the Doppler echocardiography (VINNO 6 VET, VINNO, China). The transthoracic 2D M-mode echocardiographic system was used to obtain M-mode tracings. The left ventricle internal dimension systole (LVIDs), left ventricle internal dimension diastole (LVIDd), ejection fraction (EF), and fractional shortening (FS, FS = (LVIDd − LVIDs)/LVIDd × 100%) were measured in mice anesthetized with 1.5% isoflurane.

To determine the infarct volume, the heart samples were fixed in 4% paraformaldehyde for 48-72 h and embedded in paraffin. 5 *μ*m-thick slices sections were stained with Masson's trichrome. The Image-Pro Plus analysis software was used to analyze the infarct ratio which was expressed as the volume fraction of collagen (CVF% = collagen area/tissue total area × 100%).

### 2.3. Cell Culture and Treatment

H9C2 cells (Chinese Academy of Sciences Cell Bank, Shanghai, China) were cultured in high glucose DMEM (GIBCO, USA) supplemented with 1% cyan streptomycin double-antibody and 10% (*v*/*v*) FB Sat 37°C under a 5% CO_2_ atmosphere. For the present study, H9C2 cells were incubated overnight to reach 70-80% confluence at 37°C before experimentation. H9C2 cells were treated with H_2_O_2_ at a concentration of 100 *μ*M for 4 hours to construct an apoptosis model [26]. AMPK was activated after exercise [27], so AICAR, an AMPK agonist, was used to simulate the exercise effect of H9C2 cells. Furthermore, H9C2 cells were treated with recombinant human FGF21 (rhFGF21, 75 ng/ml, 15 h; Selleck, USA), FGFR1 inhibitor (PD166866, 100 ng/ml, 15 h, Selleck Chemicals) [8], PI3K inhibitor (LY294002, 10 *μ*M, 1 h, Selleck Chemicals), AICAR (1 mM, 1 h, Selleck Chemicals) [28], and lentiviral vectors carrying *alcat1* gene (ALCAT1 OE, MOI = 1; Brain VTA, China).

### 2.4. Western Blotting Analysis

Proteins were extracted from myocardial tissues or cultured H9C2 cells using RIPA buffer containing protease inhibitors and phosphatase inhibitor cocktail, and protein concentrations in the lysate were determined using the BCA protein assay kit (Jiancheng Biotech, Nanjing, China), resolved by SDS-PAGE (30-50 *μ*g of protein per sample), and transferred onto polyvinylidene difluoride (PVDF) membranes. The blots were blocked with 5% BSA for 2 h, and the membranes were incubated with primary antibodies diluted in 5% BSA at 4°C overnight. The primary antibodies used in the present study were as follows: FGF21 (1 : 1000, Abcam), FGFR1 (1 : 1000, Abcam), ALCAT1 (1 : 1000, Abcam), p-PI3K(1 : 1000, Cell Signaling), t-PI3K (1 : 1000, Cell Signaling), p-AKT (1 : 1000, Cell Signaling), t-AKT (1 : 1000, Cell Signaling), t-IRE1*α* (1 : 1000, Cell Signaling), p-IRE1*α*(1 : 1000, Cell Signaling), t-JNK(1 : 1000, Cell Signaling), p-JNK(1 : 1000, Cell Signaling), CHOP(1 : 1000, Cell Signaling), GRP78 (1 : 1000, Bioworld), c-caspase3 (1 : 1000, Cell Signaling), Bcl-2 (1 : 800, Cell Signaling), Bax (1 : 500, Bioworld), SOD2 (1 : 1000, Cell Signaling), and GAPDH (1 : 5000, Genetex). After the membrane incubation with corresponding HRP-conjugated secondary antibodies (1 : 5000, Jackson), the western blotting bands were performed by image processing and analysis after being visualized with enhanced chemiluminescence reagents under the Gel Imaging System, quantified with the ImageJ software.

### 2.5. TUNEL Assay

Apoptosis assay was performed using a One-Step TUNEL apoptosis kit (Beyotime, China) according to the manufacturer's instruction. 20 *μ*g/ml of DNase-free protease K was added to each sample of paraffin section of myocardial tissue and incubated in dark for 30 minutes at 37°C temperatures. In the cell experiment, cells were plated on glass coverslips at a density of 1 × 10^5^ cells/well, which were fixed in 4% formaldehyde for 30 min and then permeabilized in 0.3% Triton X-100 for 10 min. Conventional paraffin sections and the cell-loaded sections were labeled with dUTP and TDT enzymes in a humidified box at 37°C for 1 h, and the antifluorescence-quenched sealing agent was used to sealing the film. The red positive particles were observed by a fluorescence microscope.

### 2.6. Kit Test of Oxidative Stress

We used the assay kits of glutathione peroxidase (GSH-Px), catalase (CAT), total superoxide dismutase (T-SOD), reduced glutathione (GSH), and malondialdehyde (MDA) (Jiancheng Biotech, Nanjing, China) to detect the enzyme activities of GSH-Px, CAT, and T-SOD and the contents of GSH and MDA, which determine the level of oxidative stress in myocardial tissue and cells. The specific experimental method is implemented according to the instructions.

### 2.7. Statistical Analysis

The percentage of myocardial collagen fibers were analyzed by the Image-Pro Plus 6.0 software (Media Cybernetics, Bethesda, MD, USA), and western blotting results were analyzed and processed by the ImageJ software (Wayne Rasband, National Institutes of Health, USA). All the experimental data were analyzed and processed by the SPSS 21.0 software (IBM Company, USA), and the results were expressed by mean ± SD. Histogram was drawn by GraphPad Prism 8.0 (GraphPad Software, La Jolla, CA, USA). Statistical differences between the groups were evaluated using the one-way analysis of variance (ANOVA) and post hoc least significant difference (LSD) multiple-comparison test. Differences were considered statistically significant at ^∗^*p* < 0.05 and ^∗∗^*p* < 0.01.

## 3. Results

### 3.1. The Cardioprotective Effect of Exercise in the Heart Was Significantly Reduced in the Post-MI *fgf21*^−/−^ Mice

**Figure 1:**
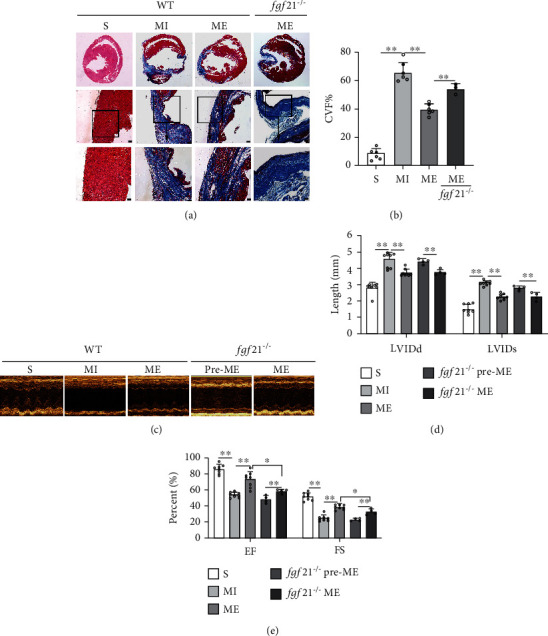
The protective of aerobic exercise on the MI heart in WT and *fgf21^−/−^* mice. (a) The myocardial infarct size was measured with Masson's trichrome staining. The images show that the fibrosis marker was stained in blue, whereas cardiac muscle fibers in red and nuclei in dark brown. Above: original magnification ×4; middle: original magnification ×10; below: original magnification ×20. Scale bar: 10 *μ*m. (b) Quantification of collagen volume of fraction (CVF%). (c) Echocardiography. (d) Left ventricular end diastolic diameter (LVIDd) and left ventricular systolic diameter systole (LVIDs). (e) Left ventricular ejection fraction (EF) and left ventricular short axis (FS). CVF: collagen volume fraction; EF: ejection fraction%; FS: fractional shortening%; LVIDd: left ventricular internal diameter diastole; LVIDs: left ventricular internal diameter systole. S: sham group; ME: MI + aerobic exercise group. Data presented are means ± SD. One-way ANOVA with post hoc LSD multiple-comparison test. ^∗^*p* < 0.05, ^∗∗^*p* < 0.01.

In our previous study, we found that the improvement of myocardial function by exercise was related to the expression of FGF21. To further confirm the role of FGF21 on cardioprotection of aerobic exercise in mice with MI, we prepared *fgf21^−/−^* mice. We quantified the collagen volume of fraction (CVF%) in the heart sections with Masson's trichrome staining. In WT mice with MI, the myocardial tissue was replaced by collagen, CVF% was significantly increased compared with the S group (*p* < 0.01, Figures 1(a) and 1(b)). Aerobic exercise significantly inhibited the further expansion of the fibrotic area of the hearts exposed to MI (*p* < 0.01, Figures 1(a) and 1(b)). However, compared with the ME group in WT mice, the inhibitory effect of exercise on excessive proliferation of myocardial collagen fibers of ME group in the *fgf21^−/−^* mice was weakened (*p* < 0.01, Figures 1(a) and 1(b)). It indicates that the repression of FGF21 expression inhibited the alleviating effect of partial aerobic exercise on the morphological changes after MI.

Echocardiography was used to detect cardiac function (Figure 1(c)). In WT mice, compared with the S group, we found and analyzed that the LVIDd and LVIDs were significantly increased and EF and FS were significantly reduced in the MI group (*p* < 0.01, Figures 1(c)–1(e)). Compared with the MI group, aerobic exercise significantly enhanced EF and FS and decreased LVIDd and LVIDs (*p* < 0.01, Figures 1(c)–1(e)). However, after inhibiting the expression of FGF21, the changes of aerobic exercise on EF and FS were attenuated (*p* < 0.05, Figure 1(e)). These results showed that aerobic exercise inhibited MI-induced heart injury at least partly via the upregulation of the expression of FGF21.

### 3.2. Aerobic Exercise Activated the FGF21/FGFR1/PI3K/AKT Pathway and Inhibited Oxidative Stress and ER Stress-Induced Apoptosis in the Heart of MI Mice

**Figure 2:**
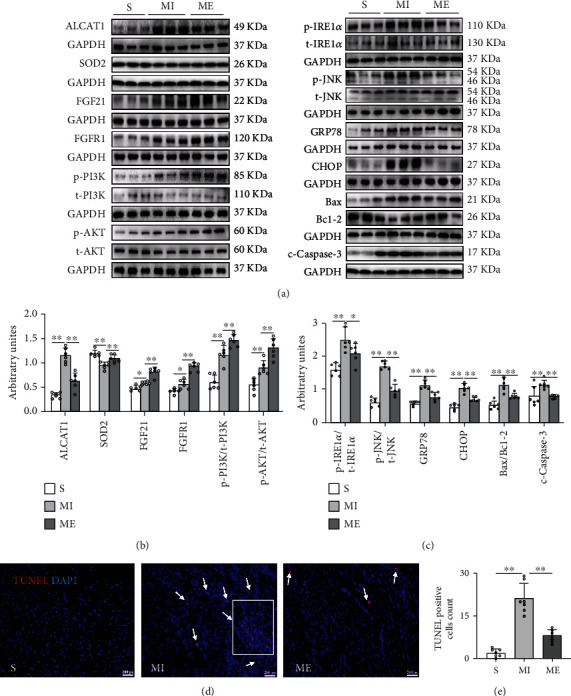
Aerobic exercise significantly inhibited oxidative stress and ER stress-induced apoptosis and activated the FGF21/FGFR1/PI3K/AKT pathway. (a–c) Western blotting images and their densitometric quantitative analysis of ALCAT1, SOD2, p-PI3K/t-PI3K ratio, p-AKT/t-AKT ratio, p-IRE1*α*/t-IRE1*α* ratio, p-JNK/t-JNK ratio, GRP78, CHOP, Bax/Bcl-2 ratio, and c-caspase3. (d) TUNEL staining: TUNEL-positive granules in red and DAPI in blue. Original magnification ×10. Scale bar: 200 px. (e) TUNEL-positive cells count. S: sham group; ME: MI + aerobic exercise group. Data presented are means ± SD. One-way ANOVA with post hoc LSD multiple-comparison test. ^∗^*p* < 0.05, ^∗∗^*p* < 0.01.

The cardiac is not only the target of FGF21 but also can autocrine FGF21 [29]. Previous studies have indicated that FGF21 inhibited oxidative stress and ER stress-induced apoptosis in myocardial ischemia injury [8, 30, 31], which was related to the activation of the PI3K/AKT pathway [4]. Exercise training boosts the level of FGF21 in the heart, liver, and circulation [12, 32, 33]. However, whether and how FGF21 participates in exercise to protect the heart from MI injury remains unclear. Firstly, in WT mice, the protein expression of FGF21, FGFR1, p-PI3K/t-PI3K ratio, and p-AKT/t-AKT ratio were increased in the MI group compared to the S group (*p* < 0.05, *p* < 0.01, Figures 2(a) and 2(b)), which was same as that of the previous research [29]. Compared with the MI group, these changed more significantly in the ME group (*p* < 0.01, Figures 2(a) and 2(b)). It was indicated that aerobic exercises upregulated the FGF21 protein expression and activated the FGFR1/PI3K/AKT signaling pathway in the heart of mice with MI.

On the other hand, ALCAT1 protein expression increased significantly, whereas SOD2 decreased significantly in the MI group compared to the S group. The six-week aerobic exercise significantly reversed these changes (*p* < 0.01, Figure 2(b)). Under the conditions of hypoxia, ER stress may lead to cardiomyocytes apoptosis [34]. And the IRE*α*/JNK pathway is a classical ER stress-induced apoptosis [35]. We observed that the p-IRE1*α*/t-RE1*α* ratio, p-JNK/t-JNK ratio, GRP78, CHOP, Bax/Bcl-2 ratio, c-caspase3 protein expression, the number of TUNEL-positive particles were significantly increased in the MI group compared to the S group. However, aerobic exercise significantly inhibited these changes (*p* < 0.05, *p* < 0.01, Figures 2(c)–2(e)). Overall, these results showed that aerobic exercise upregulated the expression of FGF21, activated the FGFR1/PI3K/AKT signaling pathway, and inhibited oxidative stress and ER stress-induced apoptosis to protect cardiac structure and function in mice with MI.

### 3.3. Knockout of *fgf21* Reversed the Effect of Aerobic Exercise on the Inhibition of Oxidative Stress, ER Stress, and Apoptosis in the Heart of Mice with MI

**Figure 3:**
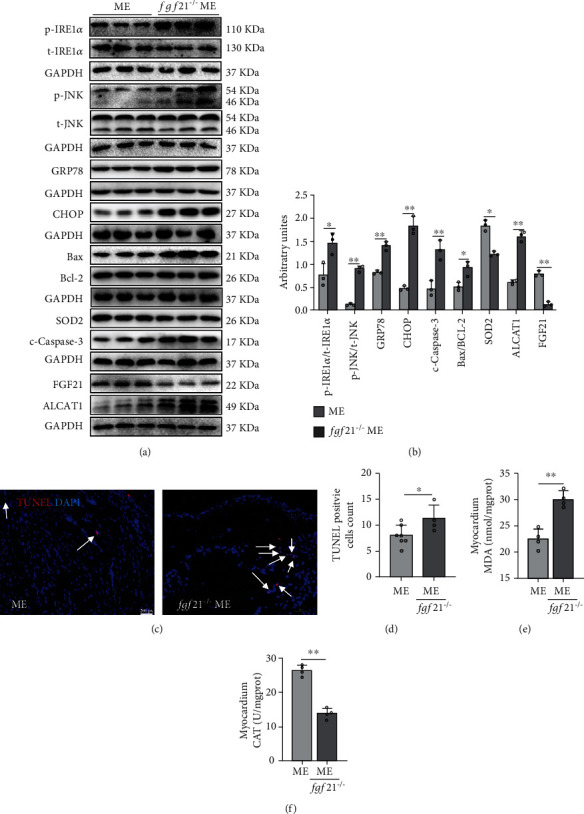
Aerobic exercise training significantly inhibited oxidative stress and ER stress-induced apoptosis partly via FGF21. (a, b) Western blotting images showed the phosphorylation levels of IRE1*α* and JNK, and the protein levels of IRE1*α*, JNK, GRP78, CHOP, Bax/Bcl-2 ratio, c-caspase3, SOD2, and ALCAT1. (c, d) TUNEL staining: TUNEL-positive granules in red and DAPI in blue. Original magnification ×10. (e) MDA content in the myocardium. (f) Enzyme activity of CAT in the myocardium. Scale bar: 200 px. ME: MI + aerobic exercise group. Data presented are means ± SD. One-way ANOVA. ^∗^*p* < 0.05, ^∗∗^*p* < 0.01.

To confirm the role of FGF21 in the inhibition of MI injury on the heart by aerobic exercise, we observed the oxidative stress, ER stress, and apoptosis in the hearts of *fgf21^−/−^* mice. Compared with the ME group in WT mice, the higher MDA, p-IRE*α*/IRE*α*, p-JNK/JNK, GRP78, CHOP, Bax/Bcl-2 ratio, c-caspase3, ALCAT1 protein expression and more TUNEL-positive particles with lower enzyme activity of CAT and SOD2 protein expressions after post-MI exercise in *fgf21^−/−^* mice were observed (*p* < 0.05, *p* < 0.01, Figures 3(a)–3(f)). These results indicated that inhibition of FGF21 expression reduced the inhibition of aerobic exercise on oxidative stress, ER stress, and apoptosis in the MI heart.

### 3.4. Aerobic Exercise and/or *alcat1* Knockout Protected the Damaged Heart in Mice with MI

**Figure 4:**
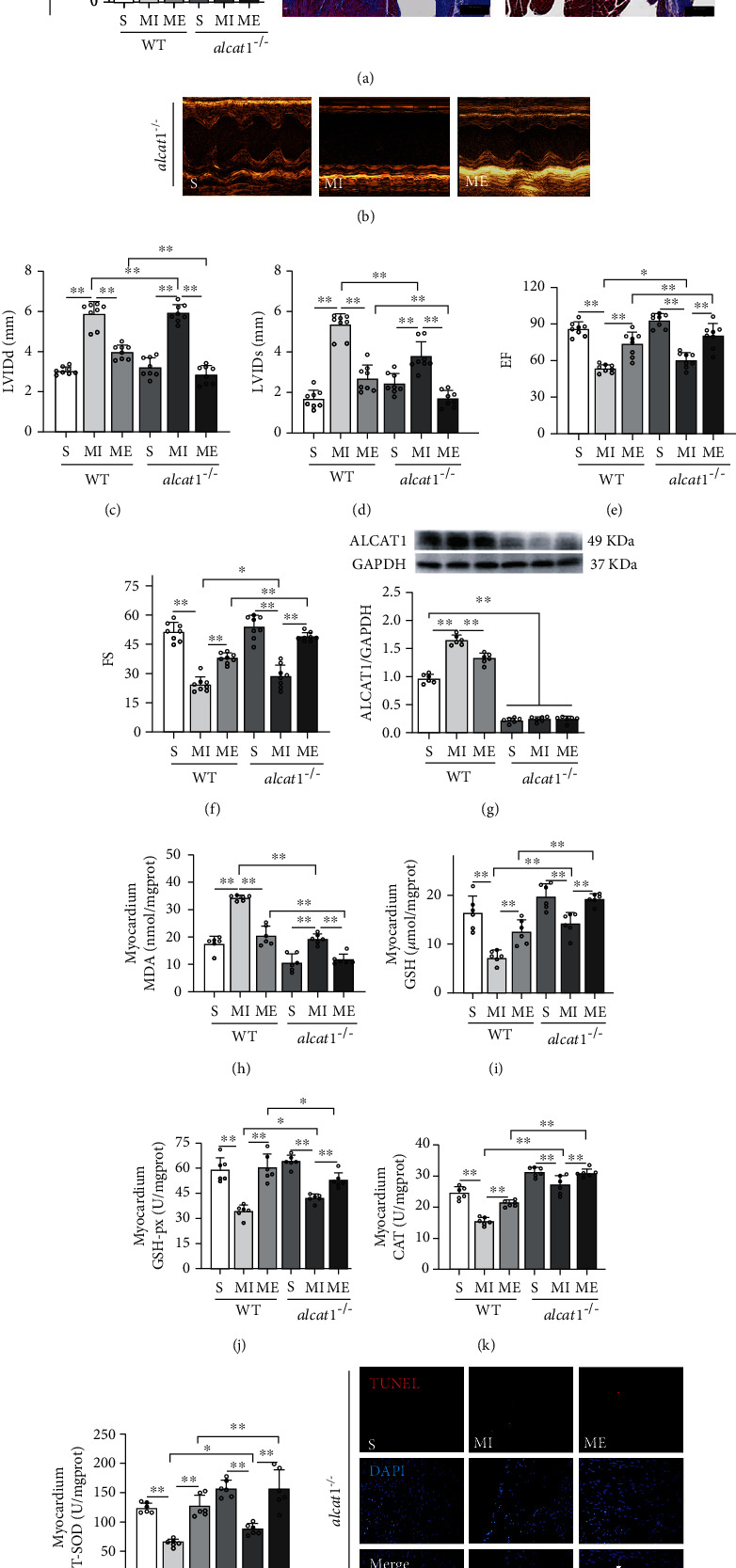
Aerobic exercise and knockout *alcat1* reduced cardiac oxidative stress and apoptosis in MI mice. (a) Myocardial infarct size was measured with Masson's trichrome staining. The images showed that the fibrosis marker was stained in blue, whereas cardiac muscle fibers in red and nuclei in dark brown. Scale bar: 200 *μ*m. Quantification of collagen volume of fraction (CVF%). (b) Echocardiography. (c) Left ventricular end diastolic diameter (LVIDd). (d) Left ventricular systolic diameter systole (LVIDs). (e) Left ventricular ejection fraction (EF). (f) Left ventricular short axis (FS). (g) Protein expression levels of ALCAT1. (h) MDA content in the myocardium. (i) GSH content in the myocardium. (j) GSH-Px activity in the myocardium. (k) CAT activity in the myocardium. (l) T-SOD activity in the myocardium. (m) TUNEL staining: TUNEL-positive granules in red and DAPI in blue. (n) TUNEL-positive cells count. (o) Protein expression levels of the Bax/Bcl-2 ratio. CVF: collagen volume fraction; EF: ejection fraction%; FS: fractional shortening%; LVIDd; left ventricular internal diameter diastole; LVIDs: left ventricular internal diameter systole. S: sham group; ME: MI + aerobic exercise group. Data presented are means ± SD. One-way ANOVA with post hoc LSD multiple-comparison test. ^∗^*p* < 0.05, ^∗∗^*p* < 0.01.

Aerobic exercise alleviates oxidative stress-induced apoptosis in kidneys of MI mice by inhibiting ALCAT1 expression [25]. However, the role of ALCAT1 in cardiovascular diseases remains unclear. We found that aerobic exercise reduced the increased expression of ALCAT1 in the hearts of mice with MI (*p* < 0.01, Figures 2(a) and 4(g)). To further examine the role of ALCAT1 on the exercise protection of MI, we used *alcat1* knockout (*alcat1*^*-/*-^) mice. We quantified the collagen volume of fraction (CVF%) in the heart sections with Masson's trichrome staining. In post-MI, the myocardial fibrosis in *alcat1^−/−^* mice was still increased which was significantly lower than that in the WT mice (*p* < 0.01, Figure 4(a)). Our results showed that six weeks of aerobic exercise training and/or *alcat1* knockout significantly reduced the fibrotic area of the hearts in MI mice and that the combined effect was better than the single effect (*p* < 0.01, Figure 4(a)). Detection of cardiac function by echocardiography (Figures 4(b) and 4(c)). In the *alcat1^−/−^* mice, LVIDd and LVIDs significantly increased, and EF and FS were significantly decreased in the MI group compared to the S group (*p* < 0.01, Figures 4(c)–4(f)). Furthermore, compared with the MI group in WT mice, the LVIDs was decreased significantly, and EF and FS were significantly increased in the *alcat1^−/−^* MI group (*p* < 0.01, *p* < 0.05, Figures 4(c)–4(f)). Compared with the WT mice in the ME group, we found that LVIDd and LVIDs significantly decreased, with the increased EF and FS in the *alcat1^−/−^* ME group. It indicated that both *alcat1* knockout and/or aerobic exercise could improve the cardiac function in mice with MI, and the combined effect of both was better.

Furthermore, the contents of MDA and GSH were significantly reduced, and the enzyme activity of the CAT, GSH-Px, and T-SOD was significantly increased in the myocardium after aerobic exercise under conditions of MI in WT mice (*p* < 0.01, Figures 4(h)–4(l)). The *alcat1*^−/−^ mice obtained similar results (*p* < 0.01, Figures 4(h)–4(l)). Furthermore, the lower MDA and the higher GSH, GSH-Px, CAT, and T-SOD were observed in *alcat1*^−/−^ mice compared to WT mice during MI (*p* < 0.05, *p* < 0.01, Figures 4(h)–4(l)). In exercise after post-MI, MDA was significantly reduced, and GSH, CAT, GSH-Px, and T-SOD were significantly increased in the *alcat1*^−/−^ mice compared to WT mice (*p* < 0.05, *p* < 0.01, Figures 4(h)–4(l)). The TUNEL assay and western blotting analysis of Bax and Bcl-2 protein levels indicated that both *alcat1* knockout and aerobic exercise significantly inhibited apoptosis in the hearts of mice with MI (*p* < 0.01, Figures 4(m)–4(o)). Thus, aerobic exercise reduced oxidative stress and apoptosis on the MI heart in mice by inhibiting ALCAT1 hyperexpression. *alcat1* knockout ameliorated MI-induced oxidative stress and apoptosis and improved the cardiac function in the MI mice. It suggests the potential role of ALCAT1 in cardiovascular disease.

### 3.5. AICAR or Exogenous rhFGF21 Inhibited H_2_O_2_-Induced Oxidative Stress and ER Stress-Induced Apoptosis in H9C2 Cells

**Figure 5:**
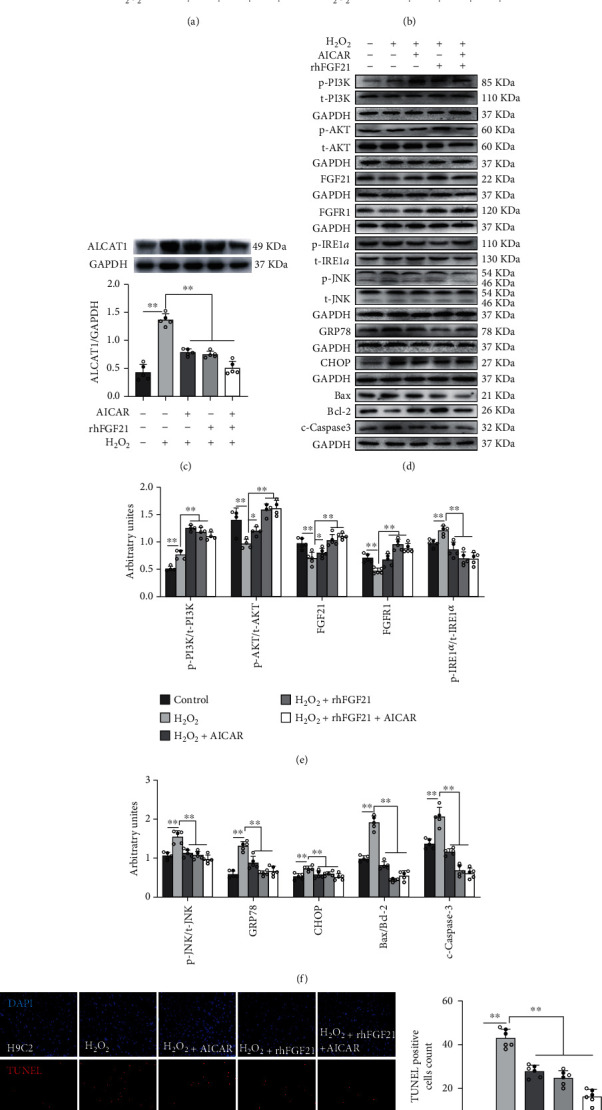
AICAR or rhFGF21 reduced the oxidative stress and ER stress-induced apoptosis by H_2_O_2_ in H9C2 cells. (a) T-SOD activity in H9C2 cells. (b) MDA in H9C2 cells. (c–f) Western blotting images and their densitometric quantitative analysis of ALCAT1, p-PI3K/t-PI3K ratio, p-AKT/t-AKT ratio, p-IRE1*α*/t-IRE1*α* ratio, p-JNK/t-JNK ratio, GRP78, CHOP, Bax/Bcl-2 ratio, and c-caspase3. (g) TUNEL staining: TUNEL-positive granules in red and DAPI in blue. (h) TUNEL-positive cells count. Data presented are means ± SD. One-way ANOVA with post hoc LSD multiple-comparison test. ^∗^*p* < 0.05, ^∗∗^*p* < 0.01.

We have found that aerobic exercise upregulated FGF21 expression, downregulated ALCAT1 expression, and inhibited oxidative stress and apoptosis in the hearts of mice with MI. To explore the possible mechanisms, we treated H9C2 cells with AICAR, rhFGF21, and H_2_O_2_ for mimicking exercise and hypoxia separately. Compared with the control group, MDA content and ALCAT1 protein expression were increased significantly, and T-SOD activity was significantly decreased in the H_2_O_2_ group (*p* < 0.01, Figures 5(a)–5(c)). All of these results were reversed by AICAR or exogenous rhFGF21 intervened. Western blotting results exhibited that the p-PI3K/t-PI3K ratio, p-IRE1*α*/t-IRE1*α* ratio, p-JNK/t-JNK ratio, GRP78, CHOP, Bax/Bcl-2 ratio, c-caspase3 protein expression, and number of TUNEL-positive particles increased significantly and p-AKT/t-AKT, FGF21, and FGFR1 protein expression decreased significantly in H_2_O_2_ group compared to the control group (*p* < 0.01, Figures 5(d)–5(h)). The rhFGF21 and/or AICAR significantly reversed these changes, the expression of p-PI3K/t-PI3K protein was further significantly increased. These data showed that exogenous rhFGF21 or AICAR activated PI3K/AKT signaling and inhibited oxidative stress and ER stress-induced apoptosis in H9C2 cells.

### 3.6. FGF21 Attenuated Oxidative Stress and ER Stress-Induced Apoptosis of H9C2 Cells by Activating the FGFR1/PI3K/AKT Pathway

**Figure 6:**
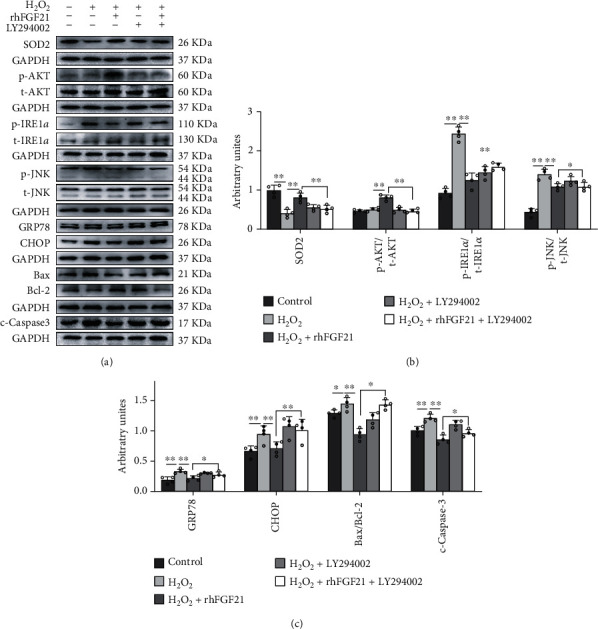
PI3K inhibitor inhibited the FGF21 overexpression-mediated oxidative stress and antiapoptotic effect on ER stress injury. (a–c) Western blotting images and their densitometric quantitative analysis of SOD2, p-AKT/t-AKT ratio, p-IRE1*α*/t-IRE1*α* ratio, p-JNK/t-JNK ratio, GRP78, CHOP, Bax/Bcl-2 ratio, and c-caspase3. Data presented are means ± SD. One-way ANOVA with post hoc LSD multiple-comparison test. ^∗^*p* < 0.05, ^∗∗^*p* < 0.01.

Our animal experiments have shown that aerobic exercise training activated the FGF21/FGFR1/PI3K/AKT pathway and inhibited oxidative stress and apoptosis on heart in post-MI mice. To further determine the underlying mechanism, we used PD166866 (an inhibitor of FGFR1) and LY294002 (a PI3K inhibitor) to treat with H9C2 cells. Exogenous rhFGF21 protected H9C2 from oxidative stress and ER stress-induced apoptosis under the H_2_O_2_ condition, but PD166866 inhibited the protective effect of rhFGF21 (*p* < 0.05, *p* < 0.01, Fig. S3). On the other hand, under the H_2_O_2_ condition, LY294002 intervention attenuated FGF21-mediated downregulation of p-IRE1*α*/t-RE1*α*, p-JNK/t-JNK, GRP78, CHOP, Bax/Bcl-2, and c-caspase3 expression, meanwhile inhibiting the upregulation of SOD2 and p-AKT/t-AKT expression (*p* < 0.05, *p* < 0.01, Figures 6(a)–6(c)). All the above data revealed that FGF21 inhibited oxidative stress and ER stress-induced apoptosis by stimulating the FGFR1/PI3K/AKT signaling pathway.

### 3.7. FGF21 Activated the PI3K/AKT Pathway to Ameliorate ALCAT1-Induced Oxidative Stress and Apoptosis

**Figure 7:**
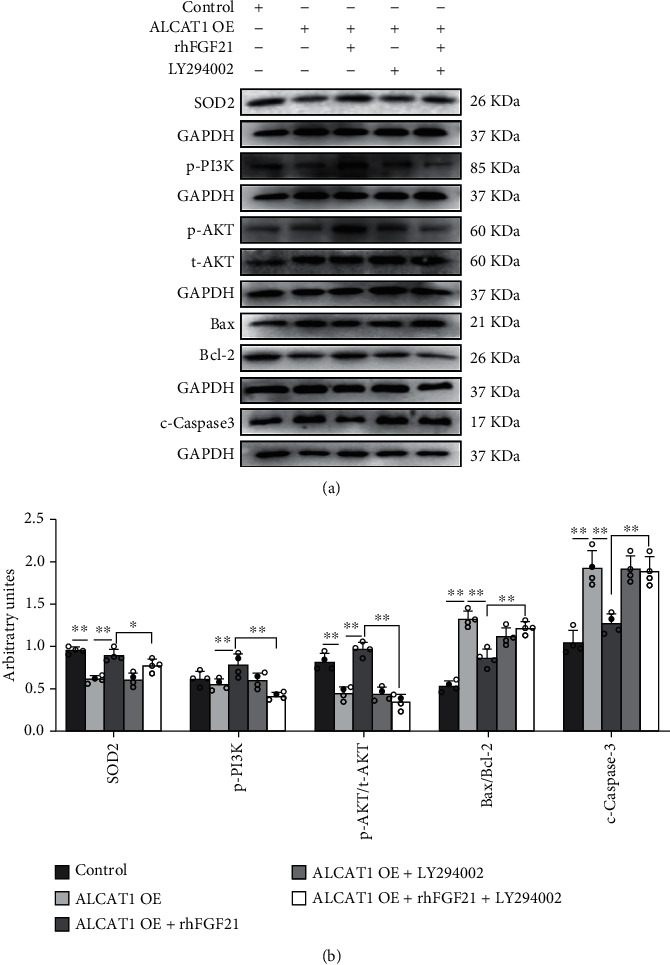
FGF21 inhibited oxidative stress and apoptosis induced by ALCAT1 overexpression through the PI3K-AKT pathway. SOD2, p-PI3K, p-AKT/t-AKT ratio, Bax/Bcl-2 ratio, and c-caspase3 protein levels after rhFGF21 or/and LY294002 intervention in H9C2 cells transfected with lentivirus containing *alcat1* gene. Data presented are means ± SD. One-way ANOVA with post hoc LSD multiple-comparison test. ^∗^*p* < 0.05, ^∗∗^*p* < 0.01.

ALCAT1 has been proved to be involved in oxidative stress and apoptosis. We speculated FGF21 might inhibit oxidative stress and apoptosis by regulating ALCAT1 expression. H9C2 cells were transfected with lentivirus containing *alcat1* gene for overexpressing ALCAT1 protein (*p* < 0.01, Fig. S4A-B). Compared with the control group, Bax/Bcl-2 ratio and c-caspase3 protein expressions were significantly increased, and the expression of SOD2 protein reduced significantly in the transfected H9C2 cells. All of these results were reversed by rhFGF21 (*p* < 0.05, *p* < 0.01, Figures 7(a) and 7(b)). It was indicated that FGF21 inhibited the hyperexpression of ALCAT1-induced oxidative stress and apoptosis in H9C2 cells.

Then, we examined whether FGF21 inhibited oxidative stress and apoptosis induced by ALCAT1 overexpression through the PI3K/AKT pathway. Compared with the control group, we found p-PI3K and p-AKT/t-AKT ratio protein expression increased significantly after rhFGF21 intervention in H9C2 cells transfected with lentivirus containing *alcat1* gene. The opposite results were observed in LY294002 and rhFGF21 cointervention group in H9C2 cells with lentivirus transfection (*p* < 0.01, Figures 7(a) and 7(b)). Therefore, exogenous rhFGF21 inhibited ALCAT1-induced oxidative stress and apoptosis by activating the PI3K/AKT signaling pathway in H9C2 cells.

## 4. Discussion

The post-MI heart undergoes extensive oxidative stress, cardiomyocytes loss, accumulation of fibrous tissue, and ultimately ventricular systolic dysfunction. Exercise-based cardiac rehabilitation is an effective treatment in attenuating post-MI apoptosis. Growing clinical consensus believes that exercise training improves myocardial oxygenation and ventricular function in patients with MI and confers sustained improvement in quality of life and reduction in cardiomyocyte apoptosis [2, 36]. In this context, our present study showed that aerobic exercise improved the cardiac structure and function through inhibiting oxidative stress and ER stress-induced apoptosis by activating the FGF21/FGFR1/PI3K/AKT pathway in post-MI heart, but *fgf21* knockout partially weakened the cardioprotective effect of aerobic exercise in mice with MI. Additionally, aerobic exercise alleviated oxidative stress and apoptosis and improved the cardiac structure and function by inhibiting the hyperexpression of ALCAT1 in the post-MI mice heart. Knockout *alcat1* alleviated oxidative stress, apoptosis, and cardiac fibrosis and improved left ventricular function in mice heart with MI. Furthermore, exogenous rhFGF21 intervention activated the PI3K/AKT pathway and significantly inhibited H_2_O_2_ or ALCAT1-induced oxidative stress and apoptosis. These results suggested that aerobic exercise can alleviate oxidative stress and apoptosis by activating the FGF21/FGFR1/PI3K/AKT pathway or inhibiting the hyperexpression of ALCAT1 and ultimately improve the cardiac structure and function of mice with MI.

FGF21 was initially found to play a role in glucose and lipid metabolism and insulin secretion (Nishimura T et al., 2000; Kharitonenkov et al., 2011; Zhang J et al., 2014). A series of subsequent clinical studies have shown that FGF21 binds to FGFR1 in the form of autocrine or paracrine in the heart [9, 37], which can be used as a predictor of cardiovascular diseases such as acute myocardial infarction and coronary heart disease and play a potential role in prevention and rehabilitation of cardiovascular diseases [38–41]. Deficiency of FGF21 leads to cardiac hypertrophy and adversely affects the ischemic heart. Studies have shown that the secretion of FGF21 in liver tissue increased during myocardial ischemia, and it played a cardioprotective effect through blood circulation [4, 42]. Further studies have found that the intervention of exogenous FGF21 significantly improved the survival of cardiomyocytes under ischemia and decreased the areas of myocardial infarction [29], whereas it inhibited the expression of FGFR1 increased the infarct area of ischemic myocardium [43]. Animal experiments have shown that both one-time acute exercise and exhaustive exercise significantly increased FGF21 expression in serum and skeletal muscle [12, 32, 44]. Clinical studies found that exercise promoted a significant increase in serum FGF21 levels in healthy adults [11, 32]. It suggested that FGF21 may play an organ protection role as an “exerkines.” However, it is not clear whether FGF21 is related to the cardioprotective effect of exercise on MI. First of all, in the WT mice, we found that after 6 weeks of aerobic exercise, the expressions of FGF21 and FGFR1 in the heart of the mice with MI were significantly increased, and the cardiac function was significantly improved. Therefore, it was believed that the improvement of cardiac function in MI hearts by exercise was related to FGF21. Then, we performed the same experiment on *fgf21* knockout mice and found that the improvement effect of exercise on the function of hearts in mice with MI was reduced. Considering this, it seems that the improvement of cardiac structure and function in mice with MI by exercise was at least partly mediated by FGF21. But its underlying mechanism remains to be further confirmed.

Oxidative stress and ER stress are highly correlated biological processes, and apoptosis induced by both of them is an important pathophysiological mechanism for cardiomyocyte injury. ER is highly sensitive to intracellular homeostasis and external stimulation. Hypoxia, DNA damage, and calcium depletion induce ER stress and eventually lead to cardiomyocytes apoptosis [36]. UPR is the initial step of ER stress, which helps to maintain ER homeostasis in an early stage. However, when UPR cannot cope with long-term or severe ER stress, the apoptosis pathway mediated by CHOP, caspase-12, and JNK is activated [35, 45]. During ER stress, JNK is phosphorylated by IRE*α*, which activates proteins from the B-cell lymphoma-2 (Bcl-2) family and caspases, which induces cell death [31, 46]. Cardiomyocytes show severe ischemia and hypoxia after myocardial infarction, which results in cellular oxidative stress, ER stress, and apoptosis [47]. Previous studies have shown that FGF21 has inhibited atherosclerosis [48], vascular calcification [49], and cardiomyocyte apoptosis by reducing ER stress [4, 8]. It was reported that overexpression of FGF21 significantly reversed the apoptosis of H9C2 cells induced by TM via inhibiting the IRE1*α*/JNK pathway [8]. There is a growing consensus that the mechanism of FGF21 in the prevention and treatment of cardiovascular diseases is related to ER stress and oxidative stress. Cardiac tissue antioxidant genes (UCP3 and Sod2) are upregulated in humans with failing hearts which may have a relation that FGF21 mRNA is upregulated [29]. In addition, FGF21 prevents type-2 diabetic lip toxicity-induced cardiomyopathy through activation of the antioxidant pathway effect in the hearts of mice [50]. FGF21 inhibits hypoxia and ER stress-induced apoptosis by activating the PI3K/AKT pathway [29, 51]. Our animal experimental study found that the FGF21/FGFR1/PI3K/AKT pathway was activated, and the oxidative stress and the expression of ER stress-related apoptosis proteins such as IRE*α*, JNK, GRP78, CHOP, c-caspase3, Bax/Bcl-2 were significantly increased in the heart of post-MI mice, whereas the phenomenon was reversed by aerobic exercise. However, the inhibitory effect of exercise on oxidative stress and ER stress-induced apoptosis were weakened after the *fgf21* gene was knocked out. To investigate its mechanisms, H9C2 cells were treated with H_2_O_2_, AICAR (AMPK agonist), FGFR1 inhibitor (PD166866), PI3K inhibitor (LY294002), and exogenous rhFGF21. We observed that AICAR and rhFGF21 promoted the secretion of FGF21 and FGFR1, activated the PI3K/AKT pathway, and inhibited oxidative stress and ER stress-induced apoptosis in H9C2 cells under the H_2_O_2_. Furthermore, the rhFGF21 intervention-mediated effect of cardioprotective was reversed by PD166866 and LY294002. Collectively, these results indicated that the protective effect of aerobic exercise on inhibiting oxidative stress and ER stress-induced apoptosis via elevating FGF21 expression in the post-MI heart was mediated, at least in part, by the FGFR1-PI3K/AKT signaling pathways.

Cardiolipin (CL) is the only phospholipid in mitochondria and rich in linoleic acid and is concentrated near the reactive oxygen production site of the mitochondrial inner membrane. CL is highly sensitive to oxidative damage, participates in the regulation of mitochondrial function and oxidative stress, and is oxidized in the early process of apoptosis [13–15]. ALCAT1, as a membrane protein with 414 amino acids, is located in the endoplasmic reticulum and mitochondrial membrane, which is very important in the process of CL remodeling. ALCAT1 catalyzes CL pathological remodeling, which leads to ROS generation, mitochondrial dysfunction, and insulin resistance under the pathological conditions of diabetes, obesity, and cardiomyopathy [16, 18, 19]. ALCAT1 overexpression results in mitochondrial division and fusion disorders, accompanied by mtDNA deletions, while the quality of mitochondria in *alcat1* knockout mice improves significantly, which is not affected by oxidative stress-induced mitochondrial swelling and fragmentation [52]. In addition, ALCAT1-targeted inactivation prevents diet-induced obesity, NAFLD, MPTP-induced neurotoxicity, improves motor deficiency, and inhibits apoptosis [19, 20, 23]. Therefore, ALCAT1 plays an important role in the process of oxidative stress-related diseases, but the role of ALCAT1 in cardiovascular diseases has not been clarified. Exercise inhibits the expression of ALCAT1 in the skeletal muscle of mice [24] and also inhibits the expression of ALCAT1 in the kidney of mice with MI and reduces renal oxidative stress and apoptosis [25]. Aerobic exercise significantly inhibits the level of oxidative stress in the ischemic myocardium [53], but ALCAT1 whether involved in its mechanism has not been fully elucidated. Our study showed that in WT mice, the expression of ALCAT1 was significantly increased in the hearts of mice with MI, and aerobic exercise mitigated oxidative stress and apoptosis induced by MI through inhibiting the hyperexpression of ALCAT1. To further determine the role of ALCAT1, we prepared *alcat1* knockout mice and found that *alcat1* knockout significantly improved cardiac structure function by alleviating oxidative stress and apoptosis in mice with MI. In cell experiments, H9C2 cells were transfected with lentivirus containing the *alcat1* gene. Exogenous rhFGF21 intervention inhibited the oxidative stress and apoptosis induced by ALCAT1 overexpression, while PI3K inhibitor (LY294002) attenuated the inhibitory effect of FGF21. These results indicated that aerobic exercise improved cardiac structure and function of mice with MI through inhibiting oxidative stress and apoptosis by suppressing the expression of ALCAT1. Meanwhile, exogenous FGF21 inhibited oxidative stress and apoptosis of H9C2 cells induced by ALCAT1 overexpression by activating the PI3K/AKT pathway.

## 5. Conclusion

**Figure 8:**
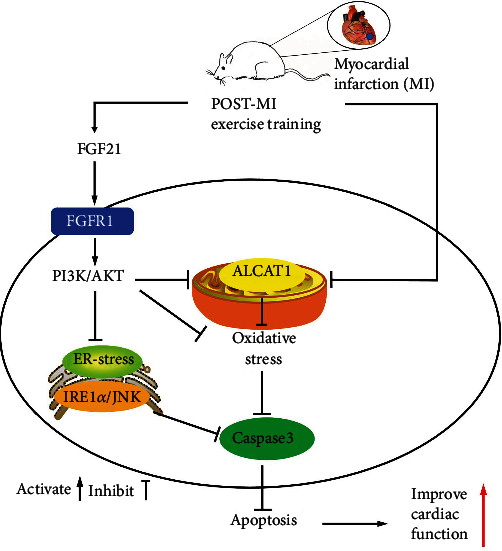
Possible mechanism of aerobic exercise in improving cardiac function in myocardial infarction mice.

In conclusion, aerobic exercise alleviated oxidative stress and apoptosis by activating the FGF21/FGFR1/PI3K/AKT pathway or inhibiting the hyperexpression of ALCAT1, which improved the cardiac structure and function of the heart in mice with MI (Figure 8). This study further elucidates the protective mechanism of exercise on the ischemic heart and suggests that FGF21 and ALCAT1 may be important molecular targets for clinical treatment or prevention of myocardial infarction.

## Data Availability

The [DATA TYPE] data used to support the findings of this study are included within the article.

## Additional Points

(1) Aerobic exercise inhibited oxidative stress and ER stress-induced apoptosis of cardiomyocytes by activating the FGF21/FGFR1/PI3K/AKT pathway in post-MI heart, and *fgf21* knockout partially weakened the protective effect of aerobic exercise on the myocardial injury in MI mice. (2) Aerobic exercise alleviated oxidative stress and apoptosis, improved cardiac function by inhibiting the hyperexpression of ALCAT1 in the heart of post-MI mice. Knockout *alcat1* alleviated oxidative stress, apoptosis, and cardiac fibrosis and improved left ventricular function in mice heart with MI. (3) Exogenous rhFGF21 activated the PI3K/AKT pathway and significantly inhibited H_2_O_2_ or ALCAT1-induced oxidative stress and apoptosis in H9C2 cells.

## Supplementary Materials

Supplementary Materials Supplementary data to this article can be found in Supplemental Files. The supplementary materials include four figures, including the identification results of mouse tail, the protein expression results after cell culture intervention, and the results of alcat1 lentivirus transfection of H9C2 cells. Fig. S1: the electropherogram of *alcat1*^−/−^ mouse tail DNA. Fig. S2: the electropherogram of fgf21 *loxp*^+/+^ mouse tail DNA. Fig. S3: FGFR1 receptor inhibitors inhibited the protective effect of FGF21 on H2O2-induced H9C2 cell injury. Fig. S4: The lentiviral vector containing *alcat1* gene was transfected into H9C2 cells.
