# Supplementary material for: The Roles of FGF21 and ALCAT1 in Aerobic Exercise-Induced Cardioprotection of Postmyocardial Infarction Mice
Source: Oxid Med Cell Longev. 2021 Nov 5;2021:8996482. doi: 10.1155/2021/8996482 (PMC8589520; doi:10.1155/2021/8996482)
Supplement: Supplementary Materials — Supplementary data to this article can be found in Supplemental Files. The supplementary materials include four figures, including the identification results of mouse tail, the protein expression results after cell culture intervention, and the results of alcat1 lentivirus transfection of H9C2 cells. Fig. S1: the electropherogram of alcat1−/− mouse tail DNA. Fig. S2: the electropherogram of fgf21 loxp+/+ mouse tail DNA. Fig. S3: FGFR1 receptor inhibitors inhibited the protective effect of FGF21 on H2O2-induced H9C2 cell injury. Fig. S4: The lentiviral vector containing alcat1 gene was transfected into H9C2 cells. [file 8996482.f1.docx]

**Enclosure:**


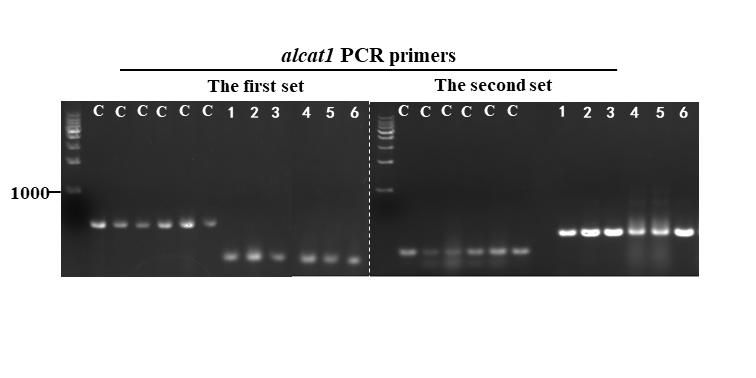


**Fig. S1. The electropherogram of *alcat1* ^-/-^ mouse tail DNA**

(C: wild-type mice)

**
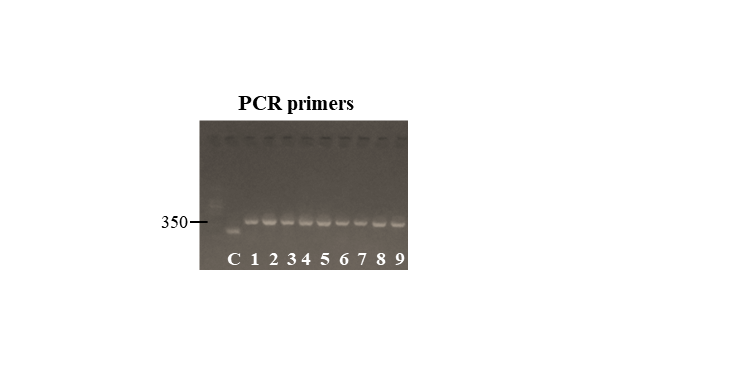
**

**Fig. S2. The electropherogram of *fgf21* ^loxp+/+^ mouse tail DNA**

(C: wild-type mice)


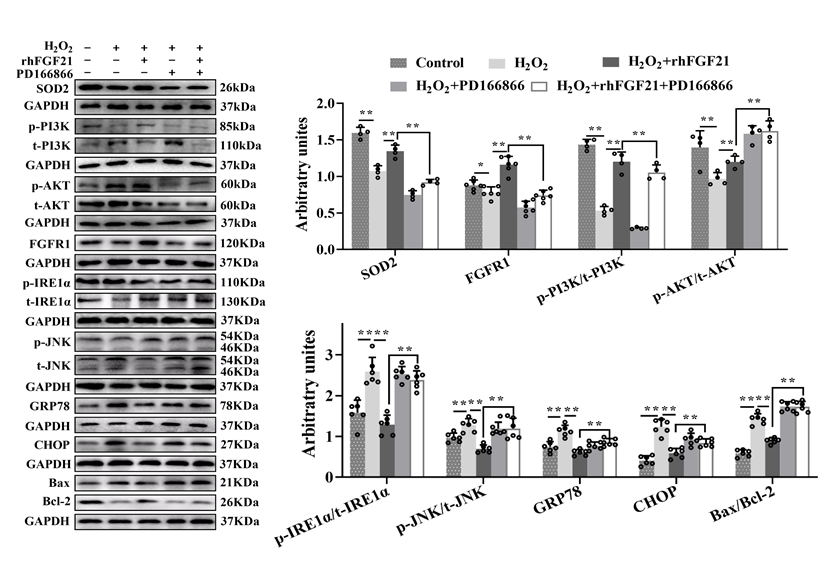


**Fig. S3.** **FGFR1 receptor inhibitors inhibited the protective effect of FGF21 on H_2_O_2_-induced H9C2 cell injury**

Western blotting images and their densitometric quantitative analysis of SOD2, p-PI3K/t-PI3K, p-AKT/t-AKT, FGFR1, p-IRE1α/t-IRE1α, p-JNK/t-JNK, GRP78, CHOP, Bax/Bcl-2 ratio. Data presented are means ± SD. One-way ANOVA with post hoc LSD multiple comparison test. **p*<0.05, ***p*<0.01.


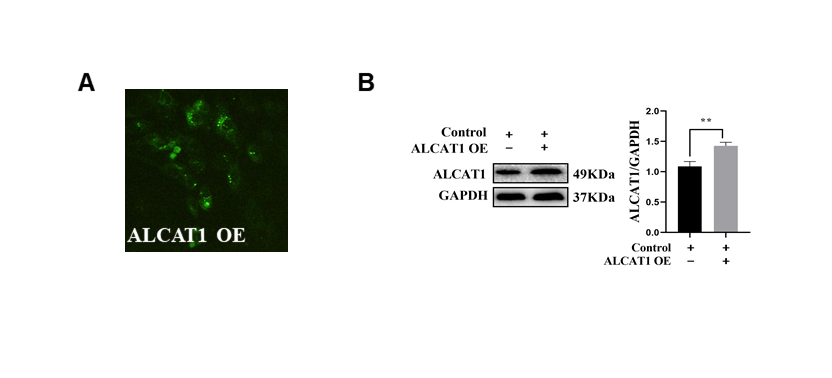


**Fig. S4. The lentiviral vector containing *alcat1* gene was transfected into H9C2 cells**

(A) 48 hours after *alcat1* gene lentivirus transfection in H9C2 cells, the green fluorescence was observed in the fluorescence microscopy. (B) ALCAT1 protein expression in ALCAT1 OE group. Data are expressed as mean ± SD. One-way ANOVA. **p*<0.05, ***p*<0.01.
